# Performance of urine cotinine and hypomethylation of *AHRR* and *F2RL3* as biomarkers for smoking exposure in a population-based cohort

**DOI:** 10.1371/journal.pone.0176783

**Published:** 2017-04-28

**Authors:** Do-Hoon Lee, Sang-Hyun Hwang, Min Kyung Lim, Jin-Kyoung Oh, Da Young Song, E. Hwa Yun, Eun Young Park

**Affiliations:** 1 Department of Laboratory Medicine, Center for Diagnostic Oncology, Research Institute and Hospital, National Cancer Center, Goyang-si, Gyeinggi-do, Republic of Korea; 2 Hematologic Malignancy Branch, Research Institute and Hospital, National Cancer Center, Goyang-si, Gyeinggi-do, Republic of Korea; 3 Department of Cancer Control Policy, Graduate School of Cancer Science and Policy, and National Cancer Control Institute, National Cancer Center, Goyang, Gyeinggi-do, Republic of Korea; 4 Cancer Risk Appraisal and Prevention Branch, National Cancer Control Institute, National Cancer Center, Goyang-si, Gyeinggi-do, Republic of Korea; 5 Carcinogenic Hazard Branch, National Cancer Control Institute, National Cancer Center, Goyang-si, Gyeinggi-do, Republic of Korea; New York University School of Medicine, UNITED STATES

## Abstract

There is a growing body of evidence demonstrating an association between smoking and DNA methylation. Accordingly, DNA methylation is now considered a promising biomarker of smoking exposure. We evaluated the relationship between methylation markers (*AHRR* and *F2RL3*) and urine cotinine as well as self-reported smoking status. DNA methylation levels of *AHRR* and *F2RL3* in blood as well as urine cotinine were measured in 330 adults (46 to 87 years of age). Pyrosequencing was performed to measure DNA methylation of *AHRR* and *F2RL3* associated with smoking exposure. The lung cancer risk associated with DNA methylation and urine cotinine was analyzed using logistic regression analysis. The *AHRR* and *F2RL3* genes were significantly hypomethylated in current smokers compared to in individuals who have never smoked. An inverse relationship was observed between urine cotinine and methylation levels. Methylation of *AHRR* and *F2RL3* distinguished current smokers from never-smokers with high accuracy. Logistic multivariate analysis showed that *AHRR* methylation is significantly associated with the risk of lung cancer (OR = 0.96, *P* = 0.011). Our study validated the smoking-associated DNA methylation markers reported in a Korean population-based cohort. In conclusion, DNA methylation of *AHRR* and *F2RL3* provided accurate measures for smoking exposure. Methylation markers reflecting the long-term effect of smoking on the risk of lung cancer showed better performance in distinguishing former smokers from never-smokers.

## Introduction

Self-reported exposure to smoke has been widely used to assess the health effects of smoking. Self-reporting, however, can be inaccurate and the amount of smoke products actually inhaled and absorbed depends on the manner of smoking [1, 2]. Serum cotinine is a better measure of smoking exposure than self-reporting methods [3]. However, it only reflects short-term exposure (half-life of cotinine in plasma has been estimated to be about 15–20 hr)[4]. Estimation of long-term smoking exposure is useful for assessing health risks accumulated through tobacco smoking[5].

Recently, long-term smoking exposure may have effects on DNA methylation patterns, which could lead to changes in gene expression and may occur in a broader context to the development or progression of various diseases [6, 7]. A recent study of multi-prospective cohorts demonstrated that hypomethylation of smoking-related genes was associated with lung cancer risk [8].

An epigenome-wide association study from the European Prospective Investigation into Cancer and Nutrition (EPIC-Turin) identified several loci including coagulation factor II (thrombin) receptor-like 3 (*F2RL3*) and aryl hydrocarbon receptor repressor (*AHRR*) that are hypomethylated in smokers compared to in non-smokers [9]. Several epigenome-wide studies have consistently shown that the methylation levels of *AHRR* and *F2RL3* were the top-ranked signals associated with tobacco smoking [7, 10] and smoking-related health risks such as lung cancer[11, 12] or cardiovascular disease[13]. Therefore, in the present study, we aimed to validate the previously identified association of the methylation levels of those genes with smoking. In addition, we evaluated the association of methylation with lung cancer risk in a Korean prospective cohort.

We quantified both urine cotinine and blood DNA methylation in a population-based cohort of older adults, and investigated the relationships between DNA methylation and smoke exposure. Furthermore, we assessed the association of both cotinine and DNA methylation biomarkers for risk of lung cancer in a Korean population-based cohort.

## Materials and methods

### Study population

A total of 110 lung cancer cases and 220 healthy controls matched with age, gender, and area of residence were randomly selected from the Korean National Cancer Center Community Cohort (KNCCC). The KNCCC is a community-based prospective cohort study designed to investigate the relationships in Korea between the risk of cancer and various environmental, lifestyle, and host factors [14]. Baseline information on age, gender, smoking status, and pack year of smokers was included in the present study. Blood and urine samples for each study participant were collected at baseline prior to development of cases and stored at −70°C or below and −20°C, respectively. These samples were used for genetic analysis and cotinine measurement. The cohort was followed from 1993 until 31 December 2012, with an average of 10.1 years of follow-up, through linkage with the Korea National Cancer Incidence Database of the Korean Central Cancer Registry and Cause of Death Database of Statistics Korea. The study participants with a cancer history before cohort enrollment were excluded in the analysis.

The study protocol was approved by the National Cancer Center Institutional Review Boards (IRB number: NCCNHS02-007; NCCNHS03-081-1; NCCNCS-07-080). Participants were provided their written informed consent to participate in this study and the IRB also approved this consent procedure.

Urinary cotinine was measured using liquid chromatography-tandem mass spectrometry (Applied Biosystems/MDS Sciex, Concord, Canada), according to a previous report [15, 16].

### Bisulfite treatment and DNA extraction

DNA samples were extracted from buffy coats using the QIAsymphony DNA Midi Kit (Qiagen, Crawley, UK). Bisulfite treatment of 2 mg of each sample was performed using the EZ DNA Methylation Kit (Zymo Research, Orange, CA, USA). The converted DNA was eluted in 50 ml of 0.1X TE buffer and pyrosequenced as previously described [17].

### PCR primer design and pyrosequencing methylation analysis

We investigated the methylation status of the *AHRR* (cg5575921) and *F2LR3* (cg03636183) promoters. PCR was performed with FastStart Taq polymerase (Roche Applied Science, IN, USA) using 2 ml of bisulfite modified DNA and 3 mM MgCl_2_, 50 mM dNTPs, and 0.2 mM primer mixture. Pyrosequencing was performed using a PSQ96MA system (Biotage, Uppsala, Sweden) according to the manufacturer’s protocol. PCR primer sequences and pyrosequencing primers are shown in (Table 1). For sequencing, the assay was validated using an internal control (non-CpG cytosine in targeted methylation sequence region). *AHRR* and *F2RL3* methylation for each sample was calculated as the average value of eight CpGs (mC/total C x 100 (%)) of the promoter CpG islands examined.

**Table 1:** PCR and pyrosequeincing primers for *AHRR* and *F2RL3*.

Gene	Forward	Reverse	Product size
*AHRR*	5′- AGGGGTTGTTTAGGTTATAGATT-3′	5′biotin- CCTACCAAAACCACTCCCAAA-3′	180
Pyrosequencing primer	5′- TTTTGAGAGGGTAGT–3′		
*F2RL3*	5′- GGGTTGGGTGTTTATTAGGT-3′	5′biotin-CAACAACAACACTAAACCATACATATA-3′	286
Pyrosequencing primer	5′- GGGGTTGTAGGTTAATGG-3′		

### Statistical analysis

The Kruskal-Wallis ANOVA test was used to compare data. Pearson tests were performed to assess any correlation between methylation values. The logistic regression model was constructed to evaluate the risk of lung cancer with methylation, urine cotinine, age, and sex. The sensitivity and specificity were calculated using receiver operative curve (ROC) analyses. Area under the curve (AUC) values were calculated for *AHRR* CG site (cg21161138) and *F2RL3* (cg03636183), and compared to those for cotinine for differentiating between never- and current smokers. All statistical analyses were performed in NCSS (NCSS, Kaysville, UT, USA).

## Results

The characteristics of the study population were described in Table 2. In this study, 320 participants were analyzed after 10 participants with no DNA samples were excluded. The median age was 65 (range 46–87) years for males (n = 223) and 65 (range 39–81) years for female (n = 97).

**Table 2:** Characteristics of the study population.

Characteristics		Case	Control
		N (%) 110 (33.3)	N (%) 220 (66.7)
Gender			
	Men	76 (69.1)	156 (70.9)
	Women	34 (30.9)	64 (29.1)
Age (year)			
	< 60	18 (16.4)	49 (22.3)
	60–69	55 (50.0)	107 (48.6)
	≥ 70	37 (33.6)	64 (29.1)
Smoking status			
	Never smokers	28 (25.5)	81 (36.8)
	Former smokers	18 (16.4)	69 (31.4)
	Current smokers	64 (58.2)	70 (31.8)
BMI (kg/m^2^)			
	Normal (< 23)	60 (54.6)	109 (49.6)
	Overweight ( ) 23 and <25)	23 (20.9)	43 (19.6)
	Obesity ( ( ) 2	23 (20.9)	63 (28.6)
	Unknown	4 (3.6)	5 (2.3)
Education level			
	Uneducated	43 (39.1)	48 (21.8)
	Elementary school or middle school	54 (49.1)	127 (57.7)
	High school or more	5 (4.6)	32 (14.6)
	Unknown	8 (7.3)	13 (5.9)
Cotinine (ng/ml) (GM ± GSD)^a^		55.5 ± 53.9	19.5 ± 23.2
Pack year among smokers (mean ± SD)^b^		37.3 ± 23.3	37.3 ± 30.2
Year follow up (mean ± SD)		5.3 ± 4.1	9.6 ± 4.0
*AHRR* methylation% (mean ± SD)^c^		55.0 ± 15.5	62.4 ± 14.6
*F2RL3* methylation% (mean ± SD)^c^,^d^		72.4 ± 8.8	75.8 ± 9.0

Abbreviations: SD, standard deviation; GM, geometric mean; GSD, geometric standard deviation

^a^ Data missing for 14, 11 and 16 participants in never, former and current smokers, respectively.

^b^ Data missing for 8 and 5 participants in former and current smokers

^c^ No DNA samples for 10 participants

^d^ Data failure for 5 participants in *F2RL3* methylation analysis

### Smoking intensity and urine cotinine levels of the study population

Smoking intensity, estimated by the self-reported smoking measured in pack-years[Median (interquartile)] among former- and current smokers was 30 (0.3–141) and 30.8 (0.9–180), respectively. The median (interquartile) cotinine concentration for current smokers was 1230.0 (645.0–1895.0) ng/ml and the median concentration of cotinine for former- and non-smokers was 2.2 (0.9–5.3) and 2.3 (0.8–4.2) ng/ml, respectively.

### *AHRR* gene and *F2RL3* gene methylation and smoking

**Fig 1:**
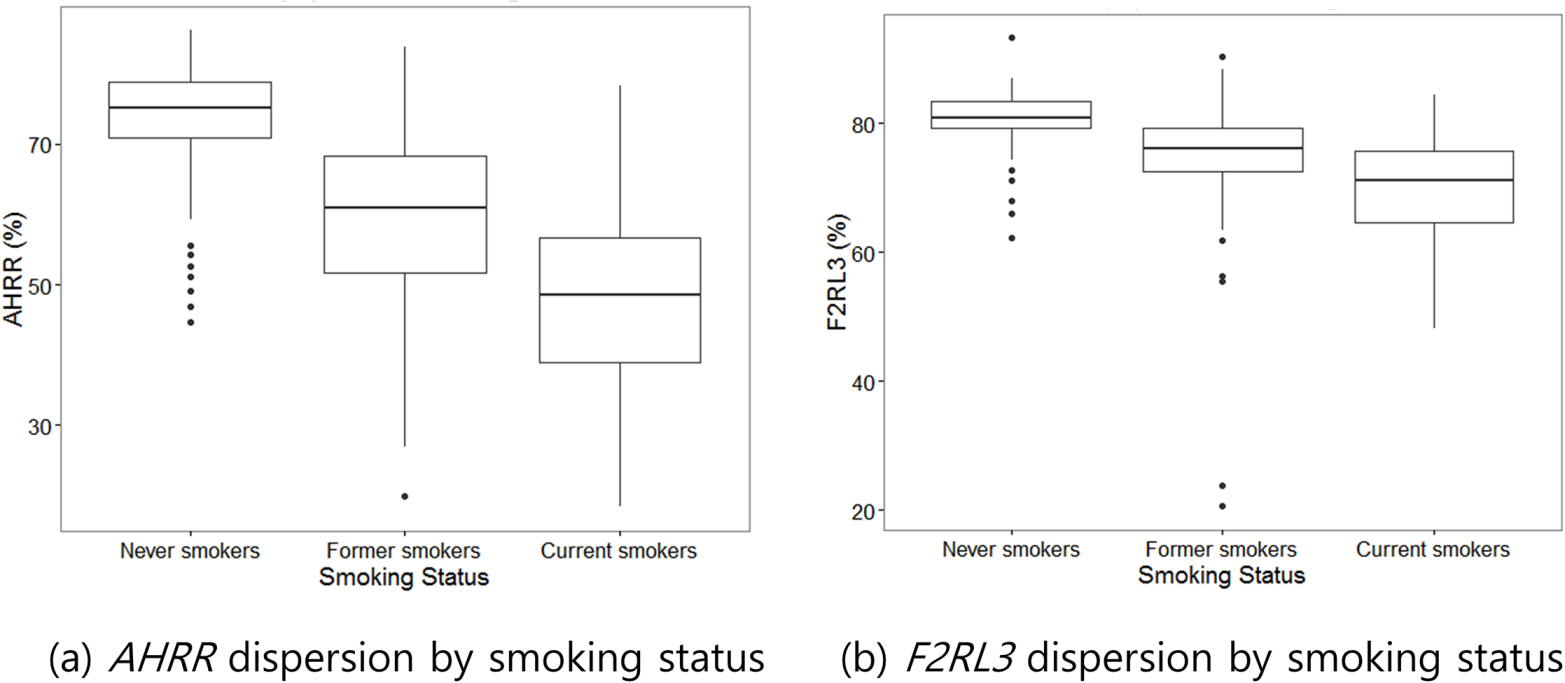
Box plots of distribution showing methylation of *AHRR* (a) and *F2RL3* (b) genes in never-, former- and current smokers.

**Fig 2:**
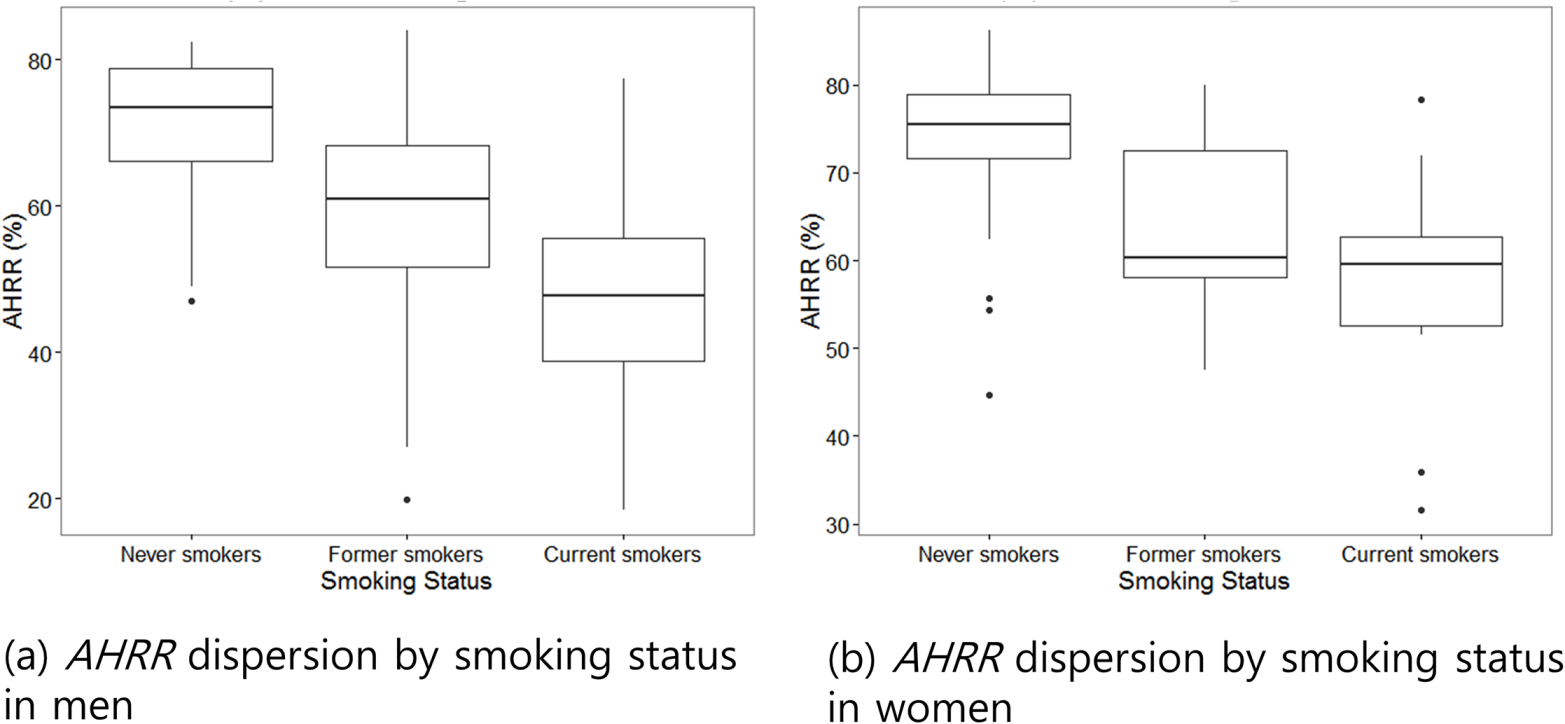
Box plots of distribution showing methylation of *AHRR* gene in never-, former- and current smokers according to gender.

**Fig 3:**
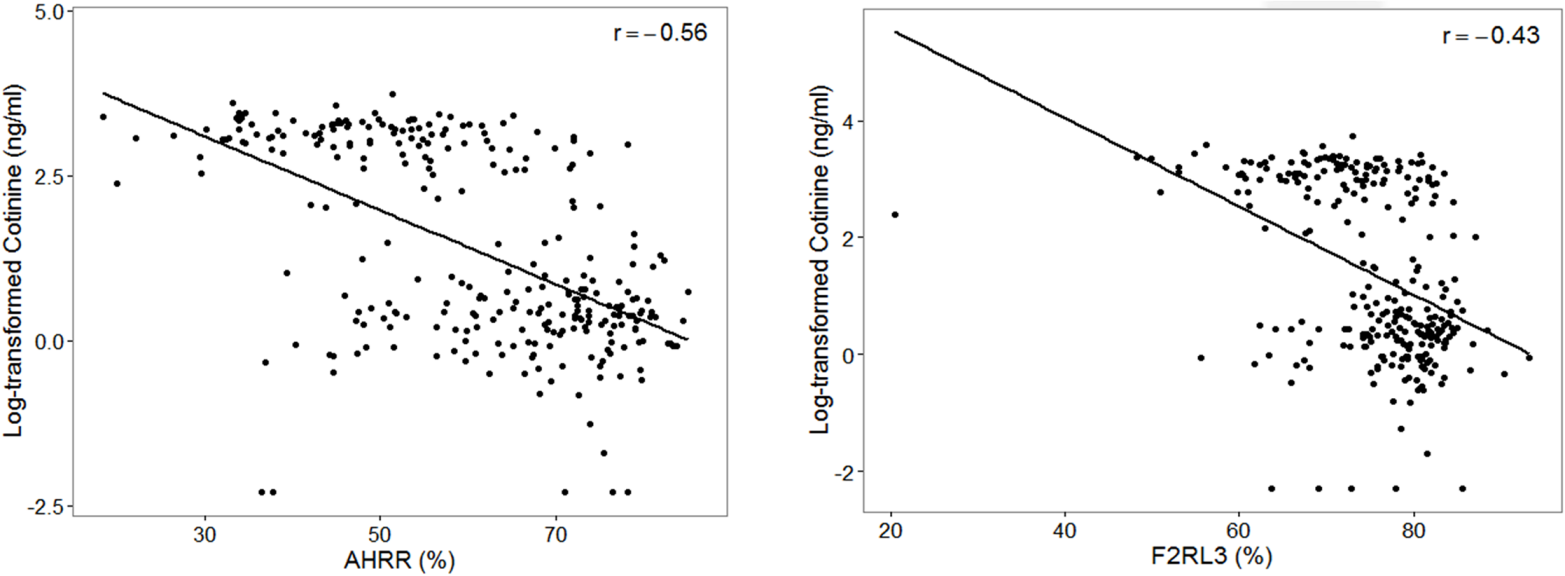
The correlation between methylation and urine continine concentration (ng/ml). The methylation (x-axis) of *AHRR* (left) and *F2RL3* (right) inversely correlated with urine cotinine concentration (y-axis).

The methylation levels of *AHRR* and *F2RL3* genes were compared according to smoking status (Fig 1). Methylation of the *AHRR* promoter in current smokers (49.1 ± 12.5%) was significantly lower than in former-smokers (59.4 ± 12.2%, *P* < 0.001) and never-smokers (73.6 ± 7.9%, *P* < 0.001, Table 3, S1 Table). *F2RL3* was also significantly hypomethylated in current smokers (70.1 ± 8.3%) compared to former- and never-smokers (74.0 ± 10.3 and 80.8 ± 4.2%, respectively, Table 3). Hypomethylation of *AHRR* (Fig 2) and *F2RL3* according to smoking status were still observed even after separating male and female study participants. An inverse relationship was demonstrated between urine cotinine and methylation of *AHRR* and *F2RL3* (r = -0.56, r = -0.43, respectively, *P* < 0.05, Fig 3).

**Table 3:** Methylation levels for *AHRR* and *F2RL3* genes as differentially methylated in current smokers vs. former- and non-smokers.

	Methylation % (mean+/- SD)			
	Current smoker (n = 130)	Former-smoker (n = 83)	Never-smoker (n = 107)	P
*AHRR*	49.1 ± 12.5	59.4 ± 12.2	73.6 ± 7.9	<0.001
Male	48.2 ± 12.2	59.1 ± 12.2	70.7 ± 10.1	<0.001
Female	57.3 ± 12.9	63.7 ± 12.2	74.6 ± 6.8	<0.001
*F2RL3*	70.1 ± 8.3	74.0 ± 10.3	80.8 ± 4.2	<0.001
Male	69.8 ± 8.2	73.8 ± 10.6	79.5 ± 5.7	<0.001
Female	72.5 ± 8.7	76.5 ± 6.7	81.3 ± 3.5	<0.001

Kruskal-Wallis ANOVA test

### Discrimination of current and former smokers vs. never- smokers by cotinine and methylation levels

#### a. Distinguishing current smokers from non-smokers

**Fig 4:**
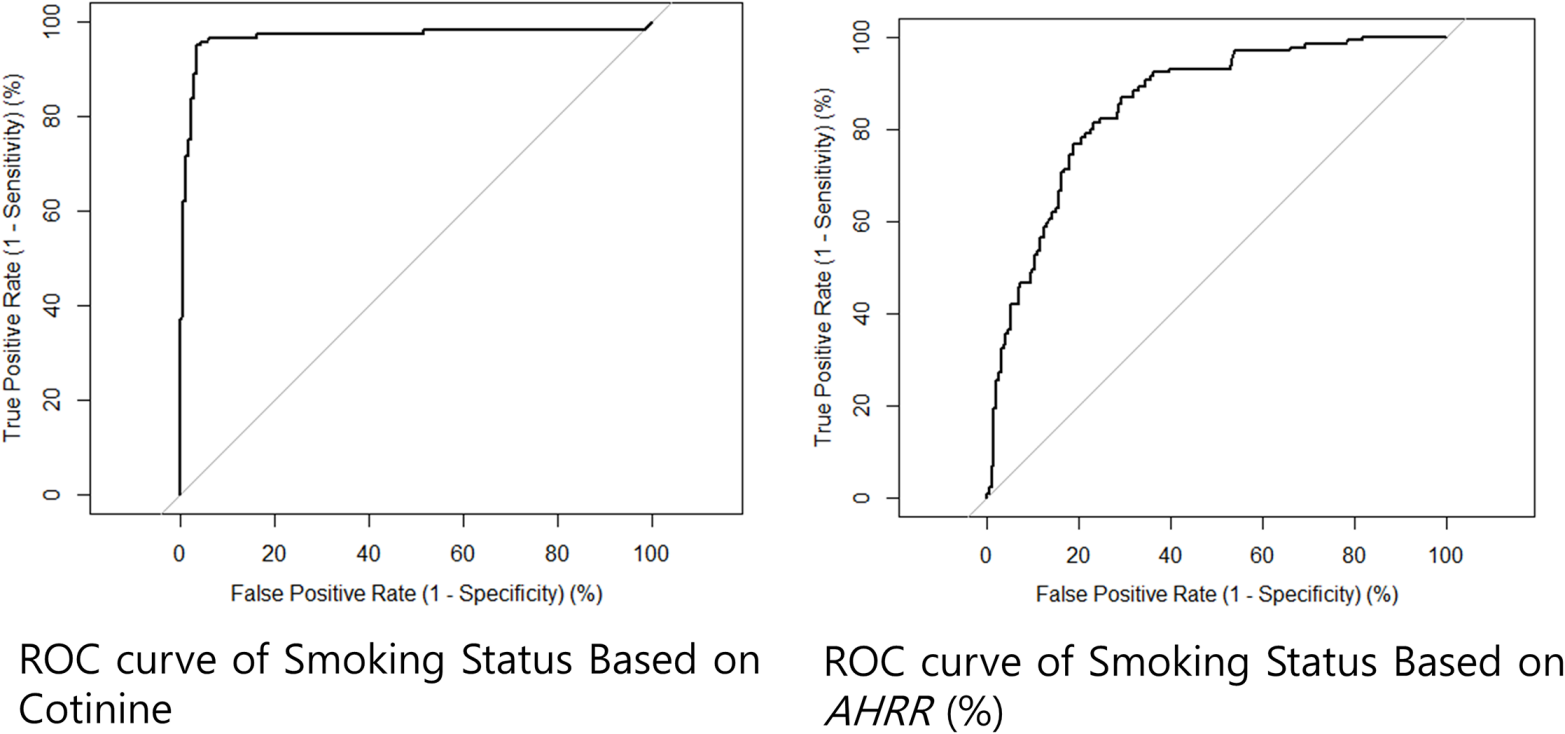
ROC analysis for predicting current smoking status. Cotinine levels above the cutoff of 201.0 ng/ml were associated with current smoking status, with high predictive value for current smokers compared to never-smokers (left, AUC = 0.974). Using a cut-off level for *AHRR* gene methylation of 60.2%, *AHRR* gene methylation also showed high performance for predicting current smokers (right, AUC = 0.942) with a sensitivity of 84.0% and specificity of 95.4%.

Table 4 indicates the AUC values based on methylation values for each genomic locus in predicting current smoker from never-smoker status, in addition to cotinine. The AUC for *AHRR* (AUC = 0.942) and *F2RL3* (AUC = 0.891) demonstrated similar ability to distinguish former from non-smokers with cotinine levels (AUC = 0.974). With a methylation cut-off of >60.2% for *AHRR*, the sensitivity was 84.0% and the specificity was 95.4% (Fig 4, Table 4). Discrimination of the performance of urine cotinine between current smokers and never-smokers (>201.0 ng/ml) showed a sensitivity of 89.5% and 100.0% specificity.

**Table 4:** The AUCs based on methylation (*AHRR* and *F2RL3*) and urine cotinine in predicting current smoker from never-smoker status.

Biomarkers (cut-off)	Sensitivity	Specificity	AUC current vs. never
*AHRR* (60.2%)	84.0	95.4	0.942
*F2RL3* (77.6%)	80.5	86.5	0.891
Urine Cotinine (>201.0 ng/ml)	89.5	100	0.974

#### b. Ability to distinguish current and former-smokers from non-smokers

Table 5 indicates the AUC values based on methylation values for each genomic locus in predicting current and former-smoker from never-smoker status. The AUCs for *AHRR* (AUC = 0.907) and *F2RL3* (AUC = 0.859) demonstrated an ability of these parameters to distinguish former from non-smokers. The sensitivity and specificity at the optimal cut-off (*AHRR* = 68.1%) were 85.4% and 86%, respectively (Table 5).

**Table 5:** The AUCs based on methylation (*AHRR* and *F2RL3*) in predicting current and former smoker from never-smoker status.

Biomarkers (cut-off)	Sensitivity	Specificity	AUC current & former vs. never
*AHRR* (68.1%)	85.4	86.0	0.907
*F2RL3* (78.8%)	80.6	79.8	0.859

### Logistic regression of methylation

Lung cancer patients had significantly more hypomethylated *AHRR* and *F2RL3* compared to controls (Table 2). Urine cotinine levels significantly increased in patients with lung cancer (Table 2).

The logistic multivariate analysis demonstrated that *AHRR* methylation was significantly associated with the risk of lung cancer (OR = 0.960, 95% CI = 0.930–0.990, *P* = 0.011). In contrast, the urine cotinine failed to show any association with the risk of lung cancer (OR = 1.019, 95% CI = 0.990–1.050, *P* = 0.2056, Table 6). Age and sex were also independent factors related to the risk of lung cancer.

**Table 6:** Odds ratios of lung cancer risk for *AHRR* and *F2RL3* methylation in multivariate analysis.

Variables	HR	95%CI	*P*
Age	1.047	1.007–1.088	0.021
Sex (male vs. female)	2.583	1.256–5.314	0.010
*AHRR* methylation	0.960	0.930–0.990	0.011
*F2RL3* methylation	1.000	0.954–1.049	0.978
Urine cotinine/creatinine (ng/mg)	1.019	0.990–1.050	0.206

## Discussion

In this study, we validated studies showing that methylation of *AHRR* and *F2RL3* showed inverse relationships with self-reported smoking status. We also identified an inverse relationship between urine cotinine and methylation markers (*AHRR* and *F2RL3*).

In this study, methylation of *AHRR* and *F2RL3* could distinguish current from never-smokers, similar to urine cotinine. Interestingly, only methylation markers were independently associated with the risk of lung cancer. In recent epigenome-wide studies of DNA methylation patterns, it has been found that there is a difference in the DNA methylation of several genes depending on the smoking status[7, 9, 10]. Fasanelli et al. showed that hypomethylation of *AHRR* and *F2RL3* genes is associated with the risk of lung cancer in 4 cohorts [8]. Our study confirmed the previous studies showing that hypomethylation of these *AHRR* and *F2RL3* genes with respect to tobacco smoking was significantly associated with lung cancer risk in a Korean prospective cohort. Logistic multivariate analysis showed that *AHRR* methylation was significantly associated with the risk of lung cancer (OR = 0.96), but cotinine was not (OR = 1.02) although univariate analysis showed that cotinine was significantly associated with the risk of lung cancer. This finding reflects the long-term effect of methylation on lung cancer development.

Multiple previous studies have investigated the relationship of cotinine levels to lung cancer and showed the relationship of cotinine levels to lung cancer risk [18–20]. Of the tobacco constituents, 4-(methylnitrosamino)-1-(3-pyridyl)-1-butanol (NNAL) and its parent compound 4-(methylnitrosamino)-1-(3-pyridyl)-1-butanone (NNK) as well as N′-nitrosonornicotine (NNN) are carcinogenic[21], but continine is a noncarcinogenic constituent. NNAL and methylation because NNAL has the longer half-life (t1/2 = 10 days to 3 weeks). The KNCC community cohort samples used in this study is derived from a population-based cohort in Korea with a wide spectrum of data to investigate other chronic diseases in the Korean population [14].

Experimentally, tobacco smoking induces “cancer-associated” epigenomic alterations in cultured respiratory epithelia [22], including lymphoblasts and pulmonary macrophages [23]. With respect to DNA methylation of peripheral blood leukocytes, the tobacoco smoke constituent metabolites enter the bloodstream and directly alter the epigenetic profile of blood leukocytes [9]. Accordingly, the biological effects of smoking could persist for extended periods following smoking cessation [24]. Thus, smoking-associated epigenetic changes could better reflect the risk of lung cancer development in former smokers than cotinine which has a relatively short half-life and was reported to be not associated with the risk of lung cancer in former smokers in large prospective study[25], although the relationship of cotinine levels to lung cancer were showed in several previous studies[18–20, 26].

A definitive inverse relation between cotinine and methylation level was recently identified [5], and we confirmed the relationship between urine cotinine and methylation markers (*AHRR* and *F2RL3*) in this study.

Although clear inverse dose-response relationships with *F2RL3* methylation intensity were reported for both current intensity and lifetime pack-years of smoking [27], we could not replicate the association between smoking intensity and biomarkers (urine cotinine and methylation of genes) in this study cohort.

Both cotinine and methylation of *AHRR* accurately distinguished current from never-smokers. However, the discrimination performance of *F2RL3* methylation (AUC < 0.900) on current smokers from non-smokers was relatively lower than that of cotinine and *AHRR* (AUC = 0.974 and AUC = 0.942, respectively). Methylation markers proved excellent for distinguishing current and former-smokers from non-smokers. Methylation markers may serve as better biomarkers for distinguishing former from never-smokers than urine cotinine because urine cotinine has a relatively short half-life and undetectable within several daysafter quitting smoking[4]. In this study, the AUCs for *AHRR* (AUC = 0.907) and *F2RL3* (AUC = 0.859) demonstrated greater ability of these parameters to distinguish former from non-smokers than cotinine levels (AUC = 0.790).

There are large differences in the duration of smoking cessation among former smokers after smoking cessation, so there are individual variations in the risk of smoking-related illness. Alterations in smoke-related methylation are largely reversible after smoking cessation within a few years of smoking cessation[28] and were found to be rapidly reversible whereas some smoking-related genes are slowly normalized or remain abnormal decades after smoking cessation[24, 28, 29]. Such slow reversible genes, potentially detectable for decades after cessation, will be useful biomarkers to precisely measure individual risk profiles of smoking-induced chronic disease such as lung cancer [30]. The expression of some genes, including *AHRR*, is not normalized to previous levels, which indicates long-term gene expression consequences resulting from DNA methylation changes of prior smoking history [9, 28]. However, methylation status of *F2RL3* is reversible, and the levels return to those of non-smokers after smoking cessation [9, 24].

*F2RL3* has significant roles in platelet activation [31] and cell signaling [32]. Recently, *F2RL3* methylation intensity showed clear dose-response relationships with smoking status and lifetime pack-years of smoking [27]. *F2RL3* methylation is a strong predictor of lung cancer risk and mortality [11].

A recent genome-wide approach revealed that *AHRR* had top-ranked signal changes in DNA methylation associated with tobacco smoking [7]. Shenker et al. showed that *AHRR* methylation is associated with smoking in WBC DNA and lung tissue [9]. The aryl hydrocarbon receptor (AHR) pathway is important to metabolize diverse biological compounds and synthetic environmental pollutants [9]. Benzopyrene and dioxin-like compounds[33] are released during smoking and are metabolized by AhR, thus releasing further carcinogenic metabolites, which may contribute to tobacco-associated inflammation and lung disease [34, 35]. The gene most strongly upregulated by smoking is *AHRR* [28]. It was demonstrated that the *AHR* pathway is required for carcinogen-induced cancer in murine models[28].

In conclusion, although there were several limitations including relatively small sample size and limited numbers of smoking-related methylation genes, this prospective study demonstrated that *AHRR* and *F2RL3* methylation levels had inverse relationships with self-reported smoking status and accurately discriminated for both current- and former- smoking. Moreover, methylation markers distinguished former smokers from never-smokers with high accuracy and significantly associated with an increased risk of lung cancer. Further large prospective studies should be performed to confirm these results. Smoking-related DNA methylations will be promising approach for noninvasive lung cancer screening or risk stratification.

## Supporting information

S1 Dataset Data. (XLSX)

S1 Table Methylation levels for *AHRR* and *F2RL3* genes as differentially methylated in current smokers vs. ever- and non-smokers in controls. (DOCX)
