# Supplementary material for: Performance of urine cotinine and hypomethylation of AHRR and F2RL3 as biomarkers for smoking exposure in a population-based cohort
Source: PLoS One. 2017 Apr 28;12(4):e0176783. doi: 10.1371/journal.pone.0176783 (PMC5409156; doi:10.1371/journal.pone.0176783)
Supplement: S1 Table — (DOCX) [file pone.0176783.s002.docx]

Table S1. Methylation levels for *AHRR* and *F2RL3* genes as differentially methylated in current smokers vs. ever- and non-smokers in controls

|  | Methylation % (mean+/- SD) | | |  |
| --- | --- | --- | --- | --- |
|  | Current smoker | Ever-smoker | Never-smoker | P |
| *AHRR* | 51.5 ± 13.4 | 60.1 ± 12.2 | 73.8 ± 7.6 | <0.001 |
| Male (n=151) | 50.6 ± 13.4 | 59.5 ± 12.1 | 73.1 ± 7.4 | <0.001 |
| Female  (n=64) | 62.7 ± 7.2 | 68.4 ± 11.4 | 74.2 ± 7.6 | <0.001 |
| *F2RL3* | 71.5 ± 8.2 | 74.0 ± 10.9 | 81.2 ± 3.7 | <0.001 |
| Male  (n=147) | 71.2 ± 8.4 | 73.8 ± 11.2 | 81.0 ± 4.2 | <0.001 |
| Female  (n=63) | 74.5 ± 4.7 | 77.8 ± 2.3 | 81.3 ± 3.5 | <0.001 |

Kruskal-Wallis ANOVA test

5 failed for *AHRR*, 10 failed for *F2RL3*
